# Granulomatous Tubulointerstitial Nephritis in a Kidney Allograft: Treatment with Interleukin-6 Receptor Antagonist Stabilises Kidney Function

**DOI:** 10.3390/jcm13123427

**Published:** 2024-06-11

**Authors:** Gabriel T. Doctor, Caroline Dudreuilh, Ranmith Perera, Anthony Dorling

**Affiliations:** Department of Transplantation, Renal and Urology, Guy’s and St Thomas’ NHS Foundation Trust, London SE1 9RT, UK; caroline.dudreuilh@gstt.nhs.uk (C.D.); ranmith.perera@gstt.nhs.uk (R.P.); anthony.dorling@kcl.ac.uk (A.D.)

**Keywords:** granulomatous tubulointerstitial nephritis, early-onset sarcoidosis, cryopyrin-associated periodic syndrome, interleukin-6 antagonist, tocilizumab, clazakizumab, kidney allograft, antibody-mediated rejection

## Abstract

Granulomatous tubulointerstitial nephritis (GTIN) attributed to early onset sarcoidosis is an ultrarare finding in an allograft kidney biopsy. We present the case of a young man with allograft dysfunction who had GTIN upon biopsy. We performed a thorough case review based on recovered records from early childhood and reassessed genetic testing results. We revised his underlying diagnosis from cryopyrin-associated periodic syndrome to early-onset sarcoidosis with wild-type *NOD2* and established a rationale to use the interleukin-6 (IL-6) receptor blocker tocilizumab (TCZ). This suppressed his inflammatory disease and stabilised kidney function. We performed a literature review related to the emerging role of IL-6 pathway blockade in kidney transplantation. We identified 18 reports with 417 unique patients treated with TCZ for indications including HLA-desensitisation, transplant immunosuppression induction, treatment of chronic antibody-mediated rejection, and treatment of subclinical rejection. Both TCZ and the direct IL-6 inhibitor clazakizumab are being studied in ongoing randomised control trials.

## 1. Introduction

We present the case of a young man with granulomatous tubulointerstitial nephritis (GTIN) in a renal allograft with declining function in the context of suboptimal oral immunosuppression use. After a thorough case review, we revised his diagnosis from cryopyrin-associated periodic syndrome to early-onset sarcoidosis (EOS) and treated his autoinflammatory condition and graft dysfunction with tocilizumab (TCZ), an interleukin-6 (IL-6) receptor antagonist, after noting that it had been previously effective in controlling his EOS. We also noted that TCZ has been increasingly used to treat chronic antibody-mediated rejection in transplantation. We performed a literature review of the emerging role of IL-6 pathway modulation in preventing and treating antibody- and cell-mediated allograft rejection.

## 2. Methods

We performed a thorough case review that we now present as a case report. To review the role of tocilizumab in kidney transplantation, we conducted a search of PubMed using the terms (((“kidney transplantation”[MeSH Terms]) OR (renal transplantation[MeSH Terms])) AND ((graft rejection[MeSH Terms]) OR (rejection[MeSH Terms]))) AND (tocilizumab[tiab] OR “interleukin-6”) AND (y_10[Filter]). We also manually searched abstracts from the American Transplantation Congress.

## 3. Case Presentation

A 20-year-old man presented with declining kidney allograft function three months after a living donation from his father. He was not fully adherent with his immunosuppression, and an allograft biopsy showed tubulitis (Banff score t2), interstitial inflammation (i2), focal arteritis (v1), and diffuse tubular injury [1]. There was mild peritubular-capillary-itis without glomerulitis (g0, ptc1, and C4d0). His total donor-specific antibody (DSA) mean-fluorescence intensity (MFI) was 13,077. In the context of low tacrolimus levels, he was treated for T cell-mediated rejection (Banff category 4 type 2A) with anti-thymocyte globulin and high-dose corticosteroids. Initially, he responded to treatment, and his DSA became undetectable.

**Figure 1:**
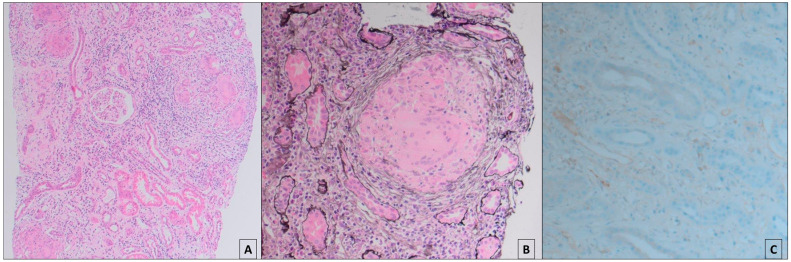
Histology from the second allograft biopsy performed 18 months post-transplantation: (**A**) ×20 magnification of renal cortex stained with haematoxylin and eosin. Illustrates widespread lymphocytic interstitial infiltrate with several granulomata. (**B**) ×40 magnification stained with periodic acid methenamine silver. Demonstrates a granuloma with multinucleated giant cells. (**C**) ×40 magnification stained with anti-C4d antibody immunohistochemistry (IHC), showing diffuse staining (C4d3) along the peritubular capillaries.

However, his renal function again worsened and he had a second allograft biopsy at 15 months. Surprisingly, this demonstrated granulomatous tubulointerstitial nephritis (GTIN), with a moderate lymphocytic infiltrate mixed with giant cells with some intracellular calcification (Figure 1A,B). There was diffuse peritubular capillary staining for C4d (Banff C4d3) (Figure 1C) but no glomerulitis, ptc-itis, or arteritis. A Ziehl–Neelson stain for acid-fast bacilli was negative, as were immunohistochemical stains for SC40, adenovirus, and cytomegalovirus. Five per cent fibrosis was noted without evidence of CNI toxicity, and accordingly, tacrolimus serum levels were consistently low. DSA testing was not performed at this stage. Although the C4d3 staining was consistent with the persistent deposition of DSA in the kidney, the multi-disciplinary team felt the absence of any microcirculatory inflammation counted against antibody-mediated rejection (AMR) being the primary pathology.

**Figure 2:**
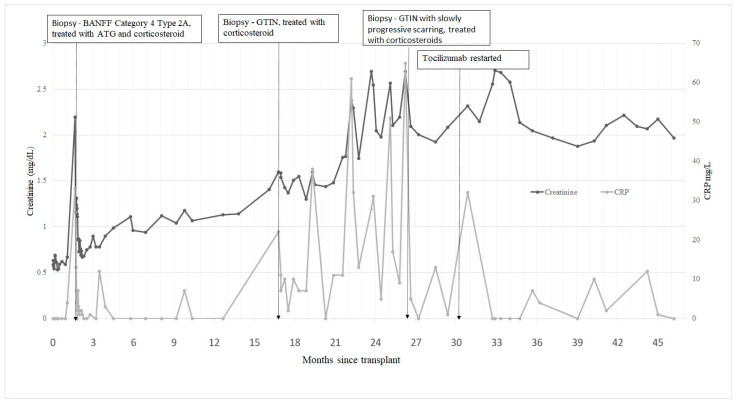
Renal function (serum creatinine) and CRP post-transplantation. Annotations illustrate the timing of the three allograft biopsies, with histological diagnoses, and the point at which anti-IL-6 treatment was re-started. (Conversion factor serum creatinine in mg/dL to μmol/L, ×88.4).

The patient was prescribed a course of prednisolone, 20 mg daily, and his tacrolimus dose was increased. Renal function again stabilised but deteriorated as the steroid dose was reduced after six months. A third allograft biopsy showed similar GTIN with giant cells and lymphocytic infiltration, identified as a mixture of B and T cells on immunohistochemistry and focal weak C4d staining of peritubular capillary walls. There was now 30–40% fibrosis. Again, glomeruli appeared normal, and there was no arteritis. Stains for acid-fast bacilli, fungi, and SV40 were again negative. A striking feature of the patient’s renal function during this stage was a correlation between serum creatinine and C-reactive protein (CRP) (see Figure 2).

In order to determine appropriate treatment, a thorough case review was performed to clarify the patient’s pre-treatment primary kidney disease.

Obtaining medical records from early childhood, we established that he presented to several centres at 2 years old after a brief febrile illness, with large, painless knee joint effusions. Growth was normal at that time. Fundoscopy showed iridocyclitis. A paediatric rheumatologist at that time made diagnoses of juvenile idiopathic arthritis (JIA) or early-onset sarcoidosis (EOS). Corticosteroid therapy was prescribed but not taken, as his parent sought homoeopathic therapy and took him abroad for alternative treatment. He re-presented at our paediatric service aged 15 years with end-stage kidney disease, with strikingly short stature and persistent, symmetrical large joint effusions. Other investigations showed CRP 35 mg/L, an erythrocyte sedimentation rate of 44 mm/h, serum angiotensin-converting enzyme (sACE) at 78 U/L (8–65 U/L) and a rheumatoid factor < 9 IU/mL. Fundoscopy showed chronic non-active papilloedema. A synovial biopsy of the right knee showed frequent, discrete, non-caseating granulomas, and stains for mycobacterial and fungal infections were negative. A renal biopsy (performed elsewhere) containing mostly capsule with a small area of cortex showed active and established tubulointerstitial nephritis without granulomata, and there was negative Congo red staining.

While awaiting initial genetic testing, a provisional diagnosis of systemic onset juvenile idiopathic arthritis (JIA) was made, and treatment commenced with corticosteroids and etanercept (an inhibitor of tumour necrosis factor-alpha) and hormones to encourage growth.

Selective exome sequencing of genes associated with paediatric inflammatory disorders was performed. No mutation was seen in *NOD2* (classically associated with early-onset sarcoidosis), but a point mutation was reported in one copy of the *NLRP3* gene (exon 3, c.1639A>T) described as a “novel or rare variant”, and a specialist team diagnosed cryopyrin-associated periodic fever syndrome (CAPS). Etanercept was stopped, and canakinumab and then anakinra (interleukin-1 [IL-1] antagonists) were trialled for eight months, unfortunately resulting in a recrudescence of knee and systemic inflammation. At that time the case was presented to a meeting as s an example of CAPS with a poor response to IL-1 therapy [2]. Tocilizumab (TCZ), an interleukin-6 (IL-6) receptor (IL-6R) antagonist, was prescribed for three years, and joint effusion improved, CRP was suppressed, and the patient gained weight. After receiving a kidney from his father, TCZ was stopped, and he was prescribed a transplant immunosuppression regimen of prednisolone, tacrolimus, and mycophenolate mofetil. The correction of uraemia led to a dramatic improvement in weight, adding 13 kg (to 47 kg) in four months.

Thus, the patient came to transplant with a diagnosis of CAPS that was unresponsive to IL-1 blockade. Following his post-transplant course and biopsy findings, we first reviewed this diagnosis. Using the gnomAD variants database, we observed that the presumed pathogenic variant in *NLRP3* identified five years earlier, though ultrarare in European ancestry populations, can be seen in 0.3% of African/African American ancestry and Latino/Admixed American populations. We established from the patient that his parents were of European and mixed Central American/African ancestry. It is unlikely that, at this frequency, this variant is pathogenic for such dramatic presentation. We recently resubmitted samples from the patient and his parents for whole-genome sequencing (Genomics England) for an extended panel of genes covering different aspects of his phenotype. No variants were identified as pathogenic.

Considering the granulomata seen in the patient’s allograft biopsies, the uncovered early history of florid effusive arthritis with granulomata seen later, and an inflammatory eye disease, we made a diagnosis of EOS with wild-type *NOD2*. We consider this, rather than CAPS, to be his underlying disease and the cause for his renal failure, and his disease went unchecked because of his parent’s preference for alternative therapies. We excluded common competing causes such as medication and infection, though repeated DSA levels would have been helpful to delineate whether he presented primarily with a recurrence of his underlying disease or with AMR in the context of an immunological predisposition to form granulomata. We believe the case for the former is strengthened by the notable co-occurrence of CRP and creatinine spikes.

In deciding how to treat the GTIN in light of the recent rejection and our new diagnosis of EOS, we considered his response to different classes of immunomodulatory medications. We noted that his tacrolimus level was frequently undetectable and hypothesised that he may be taking his oral immunosuppressants irregularly. TCZ is not a standard treatment for EOS and was first used in this case as a rescue therapy for failed IL-1 blockade as a downstream cytokine blockade in an adolescent approaching end-stage kidney disease. It had previously suppressed his inflammatory markers, and we noted the correlation between his rising creatinine and inflammatory markers post-transplant. We re-started monthly TCZ infusions. Despite poor adherence to his oral medications, he regularly attends for his infusions. There has been sustained suppression of his CRP and stabilisation of his renal graft function for the following 2 years (see Figure 2). He has not reported any infections or other adverse events. Given his stable function, the patient has declined surveillance biopsy since starting TCZ, so we cannot confirm the resolution of his GTIN, but we assume that his intrarenal inflammation has substantially resolved.

## 4. Discussion and Literature Review

This is only the fourth case report of EOS with GTIN in a renal allograft [3,4,5]; in at least one other case, tuberculosis was still considered possible [3]. We now discuss the role of IL-6 pathway modulation in preserving kidney allografts.

### 4.1. Interleukin-6 Pathway Modulation Is Powerfully Anti-Inflammatory

Our decision to use TCZ to treat the allograft dysfunction was supported by its previous effectiveness in controlling the patient’s EOS, which we considered to be a cause of the allograft dysfunction. TCZ is not a standard treatment for EOS, but there are case reports suggesting efficacy in EOS and adult sarcoidosis, supported by recent investigations identifying the potential role of IL-6 in granuloma formation [6,7]. It is widely used as maintenance therapy in other rheumatological diseases, including rheumatoid arthritis, and in Castleman Syndrome and juvenile idiopathic arthritis, in which elevated IL-6 levels contribute to disease pathogenesis [8].

IL-6, when complexed with the soluble IL-6 receptor and the ubiquitous cell-surface gp-130 receptor, is a powerful pro-inflammatory cytokine produced by both innate and adaptive immune cells, as well as many stromal cells [9]. It is a key driver of the acute-phase reaction triggered downstream of pattern recognition receptor activation and promotes the differentiation of T cells into Th17 effector cells. It is also produced by and activates B cells, inducing terminal differentiation into antibody-secreting cells with an amplifying mechanism [10]. It may contribute to germinal centre formation [11].

Chronic kidney disease, of any cause, has aspects of a systemic inflammatory disease. Higher circulating IL-6 levels correlate with an increased risk of developing CKD in the general population, and among those with established CKD, it is associated with faster progression and an increased risk of reaching end-stage renal failure, as demonstrated in two large prospective cohort studies [12,13]. IL-6 pathway activation within the kidney itself may mediate this risk. IL-6 expression is higher in both tubular and glomerular compartments of patients with kidney disease (both immune- and non-immune-mediated) than in controls [14,15]. In AKI models, kidney IL-6 levels are increased rapidly after ischaemic and nephrotoxic injury and attract neutrophils to the interstitial compartment [16]. IL-6 is also a potent activator of pro-fibrotic Th17 T cells [17]. Congenic IL-6 knockout mice have been protected from interstitial renal fibrosis after angiotensin-2 infusion [14]. The knockout mice had lower expressions of fibrosis-associated genes such as procollagen 1 and transforming growth factor B (TGFB), an effect recapitulated in wild-type mice treated with anti-IL-6 antibody [14]. While not a common therapy for tubulo-interstitial nephritis or progressive fibrosis, blockading the IL-6 pathway may attenuate a common end-pathway of kidney injury.

### 4.2. The Role of Tocilizumab in Kidney Transplantation

An extensive body of evidence links elevated IL-6 expression to solid organ transplant rejection, and a number of animal models have shown that reducing IL-6 signalling reduces both cellular and antibody-mediated rejection [9].

Our literature review, summarised in Table 1 below, identified that TCZ has been used or investigated for HLA-desensitisation, pre-transplant induction, the treatment of chronic antibody-mediated rejection, and the treatment of subclinical rejection. In total, we identified 18 reports with 417 unique patients treated with TCZ at 11 centres. The regimen is typically monthly infusions of 8 mg/kg.

#### 4.2.1. HLA-Desensitisation and Pre-Transplant Induction

We have identified four reports of 54 patients treated with TCZ in the context of HLA-desensitisation for highly sensitised patients. The first report of IL-6 receptor blockade with TCZ in the context of transplantation was in a cohort of ten patients who were unresponsive to HLA-desensitisation with intravenous immunoglobulin and rituximab who were treated with TCZ and IVIg [18]. TCZ successfully depleted donor-specific antibodies (DSA) and may have facilitated transplantation for five patients. A more recent study of TCZ monotherapy for HLA-desensitisation showed discordant results; anti-HLA antibody level MFI (mean-fluorescence intensity measured by Luminex assay) values were not reduced sufficiently to allow donor-matching to proceed [19]. More recently, Jouve and colleagues found no difference in the rate of graft survival at one year for patients desensitised with TCZ in addition to IVIg and rituximab [21].

TCZ has been trialled as an immunosuppressive induction agent. A single-centre prospective study compared the outcomes of 114 patients treated with TCZ with similar patients treated with basiliximab. There was a significant reduction in the rate of acute rejection with TCZ at 6 and 12 months and among low-risk patients at 24 months [22].

#### 4.2.2. Treatment of Allograft Rejection

The initial observation that TCZ may have reduced pathogenic anti-HLA antibodies prior to transplantation suggested a role in treating chronic antibody-mediated rejection (cABMR), which is a common cause of graft loss after transplantation. Several centres have begun to offer empirical rescue therapy with TCZ instead of, or after the failure of, standard-of-care therapies (SOC) (typically IVIg, rituximab, and sometimes plasma exchange) and have published mostly retrospective case series. We have identified twelve reports of 233 patient outcomes (with additional safety data reported on 148 patients, probably overlapping at one centre). In most cases (except [25]) TCZ was offered as a rescue therapy after the failure of SOC. Indications vary between pure chronic ABMR and mixed cellular/ABMR and in disease activity. Only one of these reports is explicitly a case–control study (TCZ *n* = 9) [28], and comparisons in these reports are mostly made against baseline DSA and biopsy parameters. These studies report heterogenous outcomes but are trying to address whether there is evidence of reduced inflammation and stabilised chronic damage on biopsy; a reduction in DSA number and MFI (high MFI has been strongly associated with graft loss) [36]; stabilised renal function; and ultimately less graft loss and better patient survival, with an acceptable adverse effect profile.

Larger studies tend to report their experiences with TCZ more favourably, which may reflect the greater experience in those centres [23,29,33]. Many report a decline in DSA MFI, including in a matched study relative to a control cohort (9 SOC + TCZ; 37 SOC) [28]. Histological follow-ups show more heterogenous outcomes: while most studies report a decrease in microvascular inflammation (Banff g plus ptc score [1]) and stabilisation of chronicity, Noble et al. (*n* = 40, with 38 follow-up biopsies) reported no statistically significant change in inflammation scores [29].

Most of these reports also assess graft and patient outcomes favourably compared with their centre’s experience with outcomes when treated with SOC: for example, at the centre with the first report of TCZ use for this indication, an initial report of 36 patients with rescue TCZ after the failure of SOC reported graft survival was 89% with a median follow-up of 3.26 years; an extension of this case series with 73 patients, followed-up for up to seven years, reported an 81% graft survival with 50% failure projected at seven years [24]. The same centre reported a comparable non-TCZ cABMR graft survival of approximately 50% at 2 years [23]. Noble et al. reported 15% graft loss at one year, and Khairallah et al. reported a 44% rejection rate soon after the discontinuation of TCZ [33]. In an interesting report on TCZ use in a paediatric cohort, only 1/25 graft loss was observed over a median follow-up of 15.8 months [31]. However, in a matched study, no difference was observed in the rate of graft loss between the two groups [28].

Safety is a key concern. Leukopenia is common and, in all studies, was a reason for temporary or permanent discontinuation. A case of infectious colitis and bowel perforation was noted in the first HLA-desensitisation study with tocilizumab [18], highlighting an increased risk of bowel perforation well-known in rheumatological literature, and which has prevented its use for treating inflammatory bowel disease [8]. Whether there is a higher burden of infectious complications than alternatives is contentious. Sethi et al. reported on safety outcomes for 148 patients treated with TCZ over 9 years at the Cedars-Sinai Hospital in Los Angeles, California, showing a similar or better safety profile to patients treated with IVIg and rituximab. Nonetheless, with TCZ, the concern with stopping treatment in the context of intercurrent infection is that there might be a rebound IL-6 surge, possibly accelerating graft damage, as Khairallah et al.’s experience suggests [33].

Despite the enthusiastic adoption of TCZ treatment in several centres, clarity is sorely needed regarding both efficacy and indication: whether it should be first-line, rescue only, or an add-on to SOC and whether residual graft function and degree of inflammation should inform use. A single-centre randomised control trial (*n* = 50) of TCZ in addition to SOC for the treatment of cABMR is currently underway in Sweden [37], which may shift the clinical equipoise over how best to treat cABMR.

#### 4.2.3. Clazakizumab, a Novel Direct IL-6 Inhibitor

As noted, an ongoing concern with TCZ is the risk of ”rebound” IL-6 pathway activation from accumulated IL-6 if treatment is stopped or interrupted, the commonest reason for which is the development of cytopaenia. This was one motivation for the development of clazakizumab (CLZ), an anti-IL6 antibody that binds to IL-6 and prevents its interaction with IL-6R. CLZ has been used for both pre-transplant desensitisation and for ABMR. In an open-label study in the context of desensitisation, patients were treated with plasma exchange, IVIg, and then CLZ (25 mg monthly for 6 months) [38]. Antibodies in all 20 patients were depleted, allowing 18 to receive an allograft.

CLZ has been studied in a small (*n* = 20), placebo-controlled randomised (1:1) trial for the treatment of late AMR [39]. While there was a slowing of the rate of eGFR decline and a significant reduction in DSA MFI, there was a notable rate of serious adverse events, including two episodes of diverticulitis requiring surgery, Coxsackie virus-associated meningitis, and recurrent pleural effusion while on treatment. The gastrointestinal tract inflammation raised further concern that this is a feature of IL-6 pathway modulation. A subsequently reported open-label, non-randomised series followed patients for up to 2.5 years [40]. This study excluded patients with any history of IBD or diverticulosis and showed a more benign safety profile, with similar stabilisation of eGFR decline. A multi-centre Phase III trial of CLZ for the treatment of chronic active AMR is currently recruiting [41].

#### 4.2.4. Prevention of Rejection

Finally, and perhaps of most direct relevance to our case, Chandran and colleagues expanded the role of IL-6 blockade to support a role in preventing cellular rejection. They presented a prospective randomised trial of 33 patients with subclinical inflammation found on routine surveillance biopsy at 6–12 months post-transplant. Patients were randomised to either standard-of-care or standard-of-care plus TCZ [35]. While follow-up was only 1 year, they found reduced inflammation scores (Banff ti scores) on repeat biopsy in the treatment arm and a change in T cell profiles towards a regulatory T cell phenotype. This points to a possible role for IL-6 blockade not just in treating but preventing rejection, perhaps synergistically with the general anti-fibrotic properties suggested above. Our own data in relation to this question would have been strengthened if the patient had consented to repeat biopsy after starting treatment with TCZ.

## 5. Conclusions

We have presented the fourth case of EOS recurrence in a transplanted kidney. Our patient had evidence of cellular rejection on his first biopsy, but subsequent biopsies showed only GTIN with progressive scarring, possibly consistent with subclinical inflammation in the context of his disease. As extensively described in young people with chronic medical conditions, our patient was not completely adherent to transplant immunosuppression. The use of IL-6 blockade as a de facto long-acting, directly observed therapy may have suppressed both cellular- and antibody-mediated alloimmune responses and contributed to the stabilisation of his graft function. IL-6 pathway modulation has been adopted in some centres for the treatment of cABMR, and while there are signals of effectiveness in a situation where the standard of care is not highly effective, broader concerns remain about safety and the effect on rebound inflammation if treatment is stopped. It is hoped that forthcoming randomised control trials will shed a clearer light on this subject.

## Figures and Tables

**Table 1:** Summary of reports on the use of TCZ in desensitisation, induction, treatment for chronic antibody-mediated rejection (cABMR), and treatment for subclinical inflammation.

Report	Number Receiving TCZ	Method Summary	Key Findings
TCZ as desensitisation therapy			
Vo et al., 2015 [18]	10	Prospective (Phase I/II), single-centre (Cedars-Sinai Medical Center, CSMC). TCZ and IVIg after failure of desensitisation with IVIg + rituximab twice.	Reduction in DSA strength. In total, 5/10 patients subsequently transplanted within a mean of 8.1 months. No ABMR on 6-month biopsy. In total, 1/10 colonic perforation and 1/10 Bell’s palsy.
Daligault et al., 2021 [19]	14	Case series (Grenoble University Hospital, GUH). Tocilizumab monotherapy first-line for desensitization.	Statistically significant reduction in DSA strength, but only 1/14 below a threshold of MFI 10,000 set as a transplantable threshold.
Moinuddin et al., 2022 (abstract) [20]	15	Single-centre (Virginia Commonwealth University, VCU) case series. Cohort: calculated panel reactive antibody(cPRA) > 80%; no transplant 1 year after SOC.	Non-statistically significant decline in Class I and Class II HLA PRA. In total, 4/15 (27%) patients were transplanted at a median of 9 months after starting TCZ.
Jouve et al., 2023 [21]	15	Prospective, non-randomised, open-label single-centre (GUH). Cohort: cPRA > 80% or DSA against target recipient. Tocilizumab + SOC compared with SOC (rituximab and plasmapheresis, *n* = 26)	In total, 7/15 in a TCZ group were transplanted (SOC rate is not reported). TCZ group had higher MFI and more numerous antibody types at baseline. Among those receiving a transplant, graft function and survival similar in both cohorts.
TCZ as induction therapy			
Abdullaev et al., 2023 (abstract) [22]	114	Single-centre (Nephrology Service Uzbekistan) randomised control, open-label study. Compared with basiliximab (*n* = 101); stratified by acute rejection risk.	Statistically and clinically significant reduction in the rate of acute rejection with TCZ at 6 and 12 months and among low-risk patients at 24 months.
TCZ as a treatment for ABMR			
Choi et al., 2017 [23]	36	Single-centre (CSMC) case series. TCZ after failure of SOC.	Significantly reduced DSA. Significantly reduced g + ptc scores and C4d deposition (*n* = 9 with post-TCZ biopsy). Graft survival was 89% with a median follow-up of 3.26 years. The centre reported a comparable non-TCZ cABMR graft survival of approximately 50% at 2 years.
Choi et al., 2019 (abstract) [24]	73	Single-centre (CSMC) case series abstract. TCZ after failure of SOC.	Extension of above. Non-statistically significant immunodominant DSA decrease; 81% graft survival; projected 50% graft survival at 7 years.
Lavacca et al., 2020 [25]	15	Single-centre (Città della Salute e della Scienza Hospital, University of Turin) case series. TCZ monotherapy, first line. Median follow-up, 20.7 months.	Six-month protocol biopsy: reduction in microvascular inflammation; stabilisation of chronicity; reduction in mean DSA MFI.; reduced rate of eGFR decline; 1/15 graft loss. Mentions 13 additional patients on TCZ who had no graft failure with a reduction in DSA MFI.
Pottebaum et al., 2020 [26]	7	Single-centre (Barnes-Jewish Hospital, WA.) case series. TCZ + SOC for acute active AMR.	A >50% reduction in immunodominant DSA was seen in 4 patients; stabilisation of renal function decline; 2/7 graft loss; 2/7 adverse infectious events.
Kumar et al., 2020 [27]	10	Single-centre (VCU) case series. TCZ after failure of SOC. Twelve months post-TCZ compared with twelve months prior per patient.	No improvement in eGFR decline and worsening of chronicity on biopsy. Higher inflammation and chronicity scores at baseline than those of Choi et al.
Massat et al., 2021 [28]	9	Single-centre (HU Toulouse Rangueil) case-control study. TCZ after failure of SOC. Cohort included mixed rejection. Compared with 37 historical SOC cohorts.	Comparatively reduced DSA MFI in TCZ vs. SOC. Greater reduction in g and ti scores. Similar graft outcomes compared with a control group and a similar rate of infection.
Noble et al., 2021 [29]	40	Single-centre (GUH) case series. TCZ for cABMR; mix of first- and second-line indications.	No statistical difference between baseline and follow-up biopsies for IFTA, g, ptc, i, t, or v scores suggests stabilisation, though there was evidence of ongoing inflammation. However, this analysis excludes those who lost their graft—15% graft loss after one year.
Sethi et al. 2021 [30]	148	Single-centre (CSMC) case–control study focusing on infectious complications.	Lower incidence rate of infections observed in tocilizumab-treated patients compared with IVIG/rituximab-treated patients (463 infections/1000 patient-years versus 730 infections/1000 patient-years).
Pearl et al., 2022 [31]	25	Single-centre (CSMC) case series—paediatric cohort; median follow-up, 15.8 months.	Stabilisation of eGFR and chronicity scores on biopsy; DSA largely stable; 1/25 graft loss.
Chamoun et al., 2022 [32]	5	Single-centre (Hospital Universitari Vall d’Hebron, Universidad Autónoma de Barcelona) case series. TCZ after failure of SOC.	Two patients discontinued after first dose; no improvement in eGFR decline; no reduction of DSA intensity.
Khairallah et al., 2023 [33]	38	Single-centre (Columbia University Irving Medical Center) case series. At least 6 months follow-up compared with 3 months prior.	No change in DSA titres; reduced interstitial inflammation scores; reduction in the rate of eGFR decline compared with before treatment; 8/18 patients rejected soon after discontinuation; 32% developed infections and 39% leukopenia.
Boonpheng et al., 2023 [34]	11	Single-centre (University of Washington Medical Center, Seattle) case series. Median TCZ duration, 12 months.	No significant difference in DSA MFI (*n* = 6); stabilised eGFR and modestly reduced donor-derived cell-free DNA, suggesting resolving kidney damage. Two moderate/severe infections.
TCZ for subclinical inflammation			
Chandran et al., 2021 [35]	16	Single-centre (University of California, San Francisco), randomised control trial for subclinical inflammation on protocol biopsy. Total of 17 in SOC group; 1 year follow-up.	Reduced inflammation scores (Banff ti scores) on repeat biopsy in the treatment arm and a change in T cell profiles towards regulatory T cell phenotype.
